# Impact of recycling on polymer binder integrity in metal injection molding

**DOI:** 10.1038/s41598-025-05577-x

**Published:** 2025-07-01

**Authors:** György Ledniczky, Ferenc Ronkay, Pál Hansághy, Zoltán Weltsch

**Affiliations:** 1 Department of Innovative Vehicles and Materials, John Von Neumann University, Kecskemet, Hungary; 2 Jászberény Campus, Eszterházy Károly Catholic University, Eger, Hungary; 3 Department of Road and Rail Vehicles, Zalaegerszeg Innovation Park, Széchenyi István University, Győr Egyetem tér 1, H-9026 Gyor, Hungary

**Keywords:** Metal injection molding, Thermal degradation, Recyclability, Polymer binders, Sustainability, Mechanical engineering, Polymers

## Abstract

Metal Injection Molding (MIM) is a manufacturing process that integrates polymer binders with metal powders to produce high-precision components, offering both material efficiency and design flexibility. This study explores the recyclability of polymer-based feedstocks used in Metal Injection Molding, specifically evaluating how repeated recycling affects the structural integrity and thermal stability of polymer binders. Given the high cost of raw materials in MIM, optimizing recyclability is essential for reducing production costs and minimizing material waste, contributing to more sustainable manufacturing practices. To assess the feasibility of repeated material reuse, the study systematically subjected molded specimens to grinding and reinjection molding over eight consecutive cycles. The effects of reprocessing were analyzed using melt flow index (MFI) measurements, differential scanning calorimetry (DSC), and thermogravimetric analysis (TGA) to track changes in polymer viscosity, thermal behavior, and degradation. The results indicate that wax precipitation during processing alters polymer viscosity and thermal stability, leading to gradual material property changes over successive recycling cycles. However, polymer degradation-induced viscosity reduction counterbalances these effects up to the fourth cycle, ensuring processability within standard injection molding conditions. The findings underscore the significance of analytical techniques in evaluating polymer binder integrity during multi-cycle reuse. Melt flow index (MFI) initially increased, peaking at the fourth recycling cycle, and then declined, while linear shrinkage rose by approximately 3% within the first three cycles before stabilizing. SEM–EDS analyses indicated around a 20% wax loss after multiple recycling cycles, significantly influencing binder rheology. Polymer binders can thus be successfully recycled up to four times while maintaining acceptable thermal and rheological properties, supporting resource-efficient and sustainable manufacturing strategies in MIM production.

## Introduction

Metal Injection Molding (MIM) is an advanced manufacturing process that combines polymer injection molding and powder metallurgy to produce complex-shaped metal parts with high precision and efficiency. The process was first developed in the United States in the 1980s and has since been used in various industries, particularly in the defense, automotive and medical sectors^1^. Nevertheless, due to considerable initial investment requirements, MIM technology proves economically less viable for small-batch production^1,2^. To begin with, it should be applied similarly to the classical injection molding technology, and then the production can be finished in steps typical of powder metallurgy. Injection molding is conducted using conventional equipment, with molds geometrically similar to those used in standard plastic injection molding processes. The most significant difference is in the raw material processed. The MIM technology uses polymer as an auxiliary material, as a large amount of metal powder is mixed into the plastic granules. The proportion of metal powder in the matrix is 90–94% by weight. For more favorable properties, additives must be incorporated into the mixture. The formulation is considered a trade secret by the manufacturing companies, but information is available in the literature on the proportions of the components, which are almost the same as those used by industrial companies^3^ to make granules from the mixed mass, which in practice is called feedstock. It is important to note that the mixture thus prepared can be used for additive manufacturing, producing small batches or prototype products^4–6^.

**Fig. 1:**
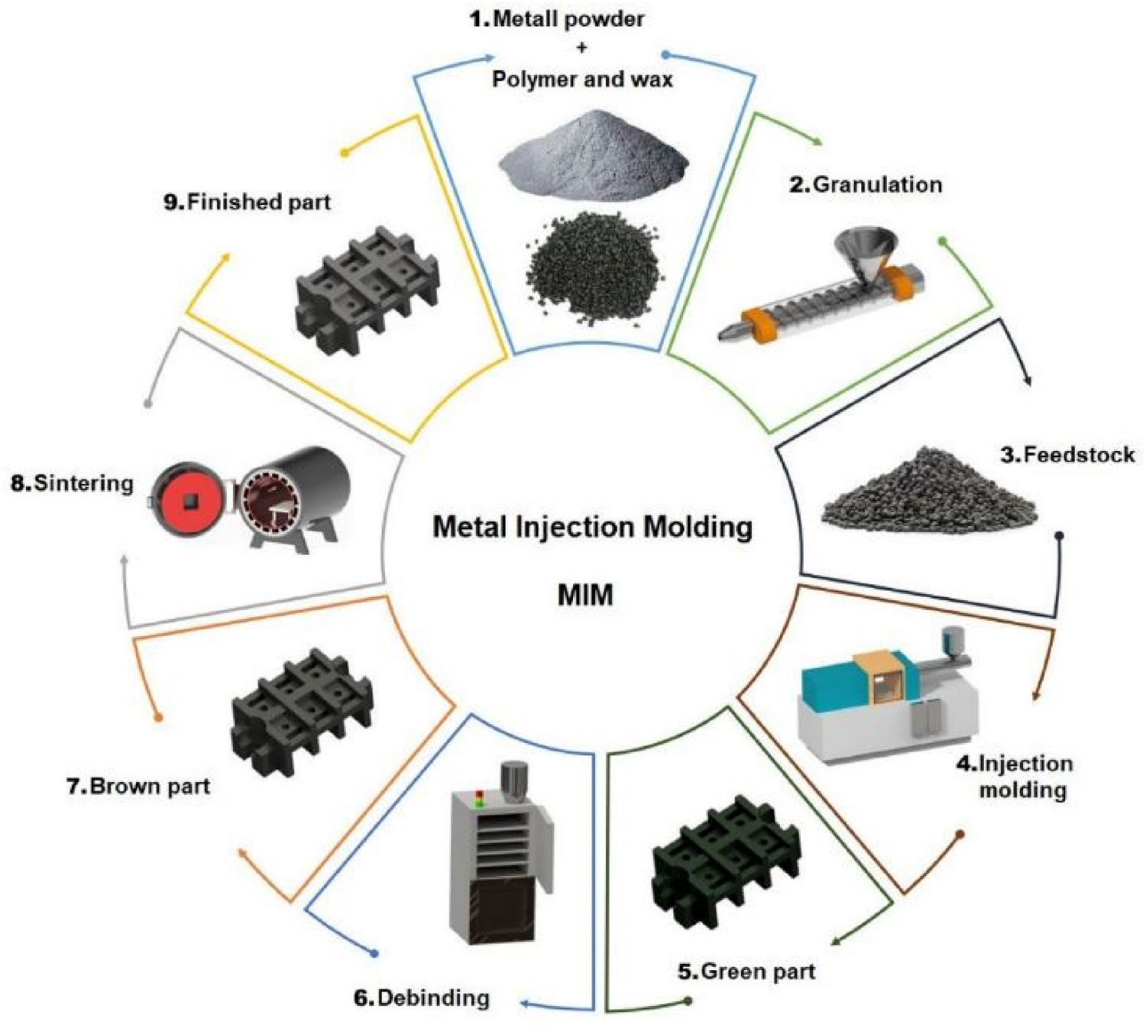
Steps of metal injection molding technology.

The process begins with the preparation and granulation of the feedstock (steps 1–3 in Fig. 1), where the appropriate amount of metal powder is blended with a polymer and wax binder system to produce homogeneous granules. The raw material is fed into the injection molding machine (step 4), where it is pressed into the mold cavity under high pressure and, after cooling and solidification, removed from the mold. This process (step 5) is referred to in the literature as the production of a so-called “green” product, which is an intermediate state and requires further technological steps. The binder plays an essential role in the bonding and molding of the metal powders when injected into the mold. The binder system contains two main components, one of which is a polymer type, and the other is a wax. In the technology presented (step 6), the binder system acts as a kind of carrier aid and is removed in the subsequent steps of the process. It is, therefore, more reasonable to use cheaper materials such as polypropylene (PP) or polyethylene (PE). Flow aid additives such as stearic acid may also be necessary to improve processability. A trichloroethylene solvent is usually used to remove the primary binder (step 7), while the secondary binder is introduced before the sintering operation^7^. The next technological step is necessary to give the component its metallic properties. The sintering of the product takes place in a high-temperature furnace, under controlled heating conditions (steps 8–9). Compared to classical powder metallurgy, MIM technology is a more complex process^8,9^. The first step is to remove the residual binder in the brown product, which is cracked off, and a small part of it is diffused into the mesh^10–12^. After the thermal degradation of the binder, the temperature has to be raised further to the sintering temperature, which is critical for the quality of the part. Significant shrinkage (~ 20%) is expected due to the removal of the binder and the bonding of the metal particles, which can cause deformation. The product is subjected to gravitational forces during sintering, whereas the bonding strength of the binder is virtually eliminated as the temperature rises so the ceramic seats must be made for products with complex geometries. In the so-called binder removal process, the binder in the green product needs to be reduced to such an extent that a porous structure is created over the entire cross-section. In practical application, this porous structure can be achieved in two steps by a so-called dual aggregate system. Several types are common, the most important being solvent, water-based and catalytic systems^13,14^. In principle, this step of the process requires the removal of about half of the binder in such a way that the solvent reacts with only one type of binder. Care must be taken to ensure that the strength can still withstand the stresses of the subsequent technological steps. After the described reduction of the aggregate, the so-called brown product is obtained, which has a porous structure, low strength and a similar size to the injection molded product.

A specific topic of interest is the effect of technological parameters on the degradation and recyclability of polymeric binders and their impact on product quality^15,16^. During the metal injection molding process, as with polymers, a quantity of unused material is created from the angus and scrap. The resulted surplus is shredded in a grinder and, depending on the type of product, partially or completely reused. Among the available literature, two publications deal with the reprocessability of MIM feedstock and its effects. Cheng et al. demonstrated that the degradation of polymer binders over successive processing cycles significantly affects injection molding performance, highlighting the importance of maintaining binder integrity^15^. Manonukul examined how different manufacturability parameters are affected by mixing different percentages of the binder with the original and recycled binder and concluded that the mechanical properties are almost the same, but shrinkage is significantly affected^16^. While previous studies examined the recyclability of MIM feedstocks highlighting viscosity and shrinkage effects, our research expands on these findings by systematically quantifying these property changes over multiple recycling cycles and clearly defining practical limits of polymer binder recyclability. Recycling of polymeric materials, including polyolefins such as polyethylene (PE) and polypropylene (PP), as well as biodegradable polymers like poly(lactic acid) (PLA), generally leads to progressive degradation in mechanical, thermal, and rheological properties. Common trends across multiple studies indicate reductions in molecular weight, crystallinity changes, and alterations in flow behavior due to polymer chain fragmentation^17–21^. For example, repeated mechanical recycling cycles typically decrease tensile strength and elongation at break while increasing melt flow indices, reflecting reduced polymer viscosity and structural integrity. Several recent studies have extensively documented these trends for various polymer systems, highlighting both negative and occasionally beneficial effects of repeated recycling, especially when considering additives or composite systems^22,23^. However, it should be noted that these observations predominantly pertain to pure polymers or general polymer composites, rather than specifically addressing polymer binders used in metal injection molding. Thus, our research aims to specifically investigate how these general trends in polymer degradation translate to the unique context of polymer binders in MIM technology. Machaka and co-authors studied the rheological properties of a proprietary aggregate and found that powder size has a significant correlation with the flow properties and injection mouldability of the MIM material^24^. A study on water-based binders investigated the difference in density and tensile strength between the injection moldability and the residual aggregate and found that the release of the binder is most affected by the molecular weight^25^. In a study, Li performed thermogravimetric, powder loading studies, MFI measurements and SEM analysis of the substrate and the effects on the dispersion and concluded that by improving the powder surface with activating agents, manufacturability was better due to enhanced binder contact^5^. Momeni et al. investigated our choice of raw material, highlighting the effect of the binder on the microstructure. They concluded that powder concentration fundamentally increases strength, but there is a limitation as it becomes heavier flowing and deteriorates the injection moldability. In addition, an increase in the PP fraction increases hardness as the residual carbon is incorporated into the metallic lattice^12,26^.

The research aims to explore the possibilities and limitations of recycling a selected raw material with a company cooperating in the research, using metal and plastic analysis methods.

## Materials and method

### Metal powder and binder

In the research, in collaboration with AFT Hungary Ltd., it became necessary to select a material that meets several criteria (wide application range, cost-effectiveness, suitability for metallographic analysis)^27^.

Based on these criteria, a low-alloy carbon-nickel steel, 4605, was selected for its favorable mechanical properties, such as high hardness (20–30 HRC in the as-sintered state and up to 50 HRC after heat treatment) and excellent wear resistance^7^ The alloy is widely used in various industrial segments, Table 1 gives the main chemical components of the alloy.

**Table 1:** Chemical composition of metal alloy expressed as w%/w%^7^.

%	Fe	C	Ni	Mo	Si
Min	Bal	0.4	1.5	0.2	-
Max	Bal	0.6	2.5	0.5	1.0

The precise binder formulations remain undisclosed due to proprietary considerations, but a similar combination can be seen in the literature. Lin, K. H. in his study investigated the fatigue hardening of an iron-nickel-based alloy. The proportions of metallic powder and binder were 55 (vol.%) and 45 (vol.%), respectively, in which the distribution of the constituents of the aggregate was as follows^29^: 55 wt.% paraffin wax, 25 wt.% polypropylene, 5 wt.% stearic acid and 15 wt.% carnauba wax.

### Method and experimental design

Objectively, the raw material should be regrinded and injection-molded several times successfully. To calculate the amount of feedstock required, it is first necessary to determine the material envelope of a generation. One generation refers to the process in which the granulated feedstock is loaded into the injection molding machine, used to produce test specimens, and then the leftover molded parts and runner systems from that cycle are collected and returned to the grinder for reprocessing.

- Injection molded test mass with the channel: 40 g,
- 3 pieces of green product taken for testing for one generation: 120 g,
- 3 pieces of green product removed for sintering: 120 g,
- Other grindings removed for analysis: 300 g,
- Total: 6 pieces + mince: 540 g.

There was no literature data available about the maximum number of reuses of the material, so we planned to process 8 generations, which is 4.4 kg of material. Considering the losses throughout, an average of 5 extra shots are required after each generation, requiring an additional 600 g of feedstock for a total of 9.2 kg.

**Fig. 2:**
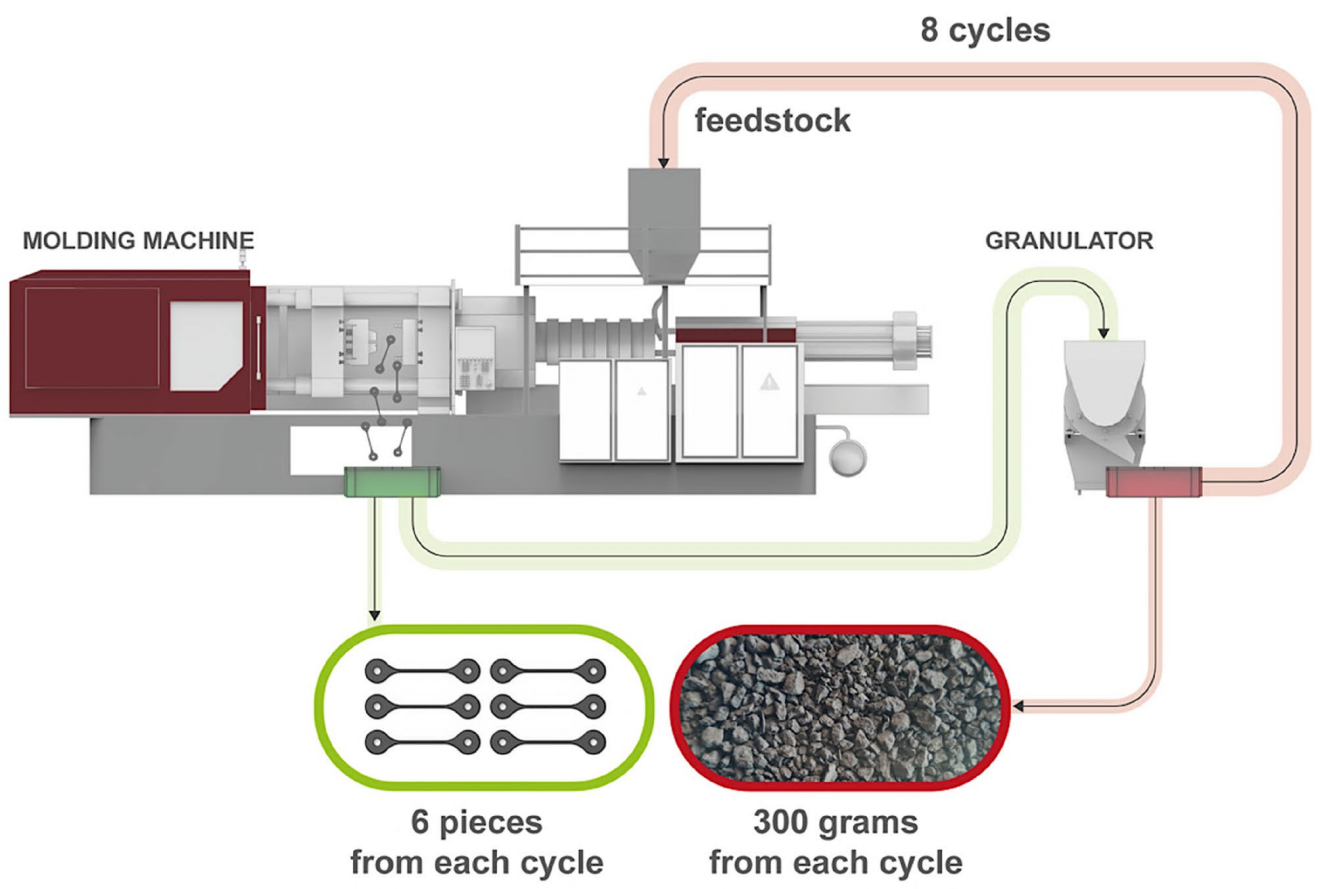
Schematic representation of the recycling process of the molded components and runner systems.

So, the trial started with 10 kg of starting material after rounding up, from which 6 samples (240 g) and 300 g of pellets were taken for each generation. At the end of the test, the amount of material determined was sufficient for 8 cycles. The schematic representation of the recycling process is depicted in Fig. 2.

### Injection molding

The experiment was carried out on a Battenfeld BA 600 CDC injection molding machine with a high wear resistance screw, where a process setting was selected by varying the parameters within the processing window.

The process parameters are presented in Table 2.

**Table 2:** Injection molding parameters set during the test.

Set parameter	Value
Injection volume	7.9—8.1 cm^3^
Injection pressure	1900–2100 bar
Holding time	3–5 s
Holding pressure	980–1070 bar
Cooling time	13 s
Mold temperature	50 °C
Melt temperature	190 °C
Cycle time	30 s
Total number of specimens produced	287 pieces
Total production time	11 h

**Fig. 3:**
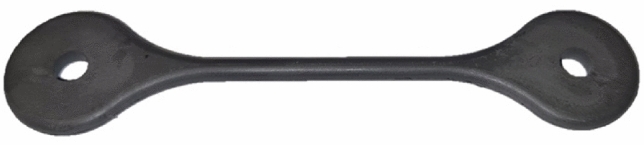
The injection molded green part.

A test specimen fabrication tool was selected to produce an ASTM standard tensile test specimen of circular cross-section^27^. Its advantage over a square cross-section specimen is that it has better flow properties, allowing a broader range of parameters to be set, which is advantageous for experiments. The geometry of the injection-molded tensile specimen is presented in Fig. 3. Tensile test specimens were injection molded during the trials, allowing for further testing and conclusions.

### Sintering process

**Fig. 4:**
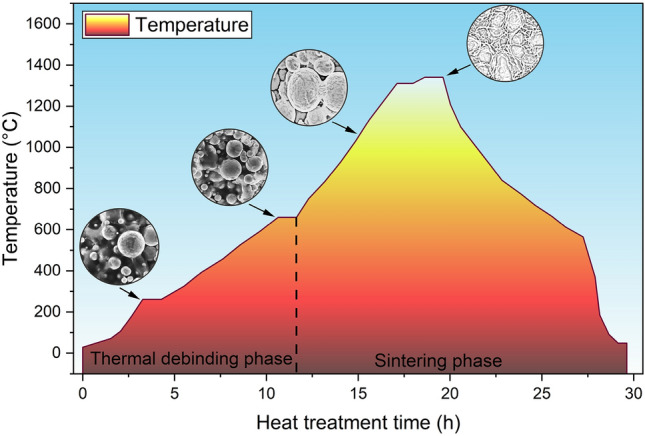
Thermal debinding and sintering diagram of the specimens.

This chapter describes the thermal binder removal and subsequent high-temperature sintering. The duration of the heat treatment was approximately 30 h, with carefully staged heating to ensure adequate removal of the residual binder, as shown in the sintering diagram in Fig. 4.

The applied heat treatment process exhibits an extended duration, significantly influencing the resulting microstructure and with the peak temperature and the corresponding heat retention time having the most significant effect on the grain structure^8^. After the right heat treatment time, the parts reach a density of 97–98%. The previously presented samples were sintered at the same time and in the same furnace, avoiding the influencing factors of the mentioned parameters.

### Test methods and equipment

The linear shrinkage was measured using a Mitutoyo PJ-H30 profile projector (ODS). To support the results obtained from this, plastic analytical methods such as thermogravimetric analysis TA Q5000 (TGA), differential scanning calorimetry TA Q200 (DSC). During the TGA analysis, the samples were heated from a starting temperature of 30 °C at a constant rate of 20 °C/min up to 800 °C, while continuously recording the mass loss. The measurements were conducted in a nitrogen atmosphere with a flow rate of 30 ml/min. DSC samples were taken and compared from the part of the test specimen near the inlet and at the end of the flow path. For the evaluation, the samples were first heated to 190 °C and annealed for 3 min to remove thermal history. They were then cooled under controlled conditions at 20 °C/min.

A tangential sigmoidal baseline was fitted at the endothermic vertices of the curves and then subtracted from the heat flow curve. Then, the multiple melting peaks were separated using the Fraser-Suzuki function and its fitting parameters were determined for each peak element. The chosen function can handle the possible asymmetry of the curves recorded during linear heating, in contrast to the traditionally used Gaussian or Lorentz functions (1):

1 $$q={D}_{K}expexp \left[-ln(2){\left[\frac{ln\left(1+2{J}_{K}\frac{T-{E}_{K}}{{F}_{K}}\right)}{{G}_{K}}\right]}^{2}\right]$$

where q [mW/g] is the specific heat flux; T [K] is the temperature; D_K_ [mW/g] is the amplitude constant; E_K_ [K] is the position constant; F_K_ [K] is the half-width constant; and G_K_ [-] is the asymmetry parameter.

The evaluation was carried out on a second heating curve of 20 °C/min. Melt Flow Index (MFI) measurements were performed using a Ceast MF10 measuring machine, with settings optimized for CP1200B-type polypropylene (PP) within the previously discussed alloy, MIM-4605. The test parameters were set to 230 °C and a load of 2.16 kg. The SEM images were taken with a Zeiss Sigma 300VP electron microscope, complemented with an energy-dispersive X-ray detector (EDS) suitable for elemental analysis. To obtain high-quality images, a combined secondary electron detector (SE) was used.

## Results and discussion

### Change of linear shrinkage and melt flow index

**Fig. 5:**
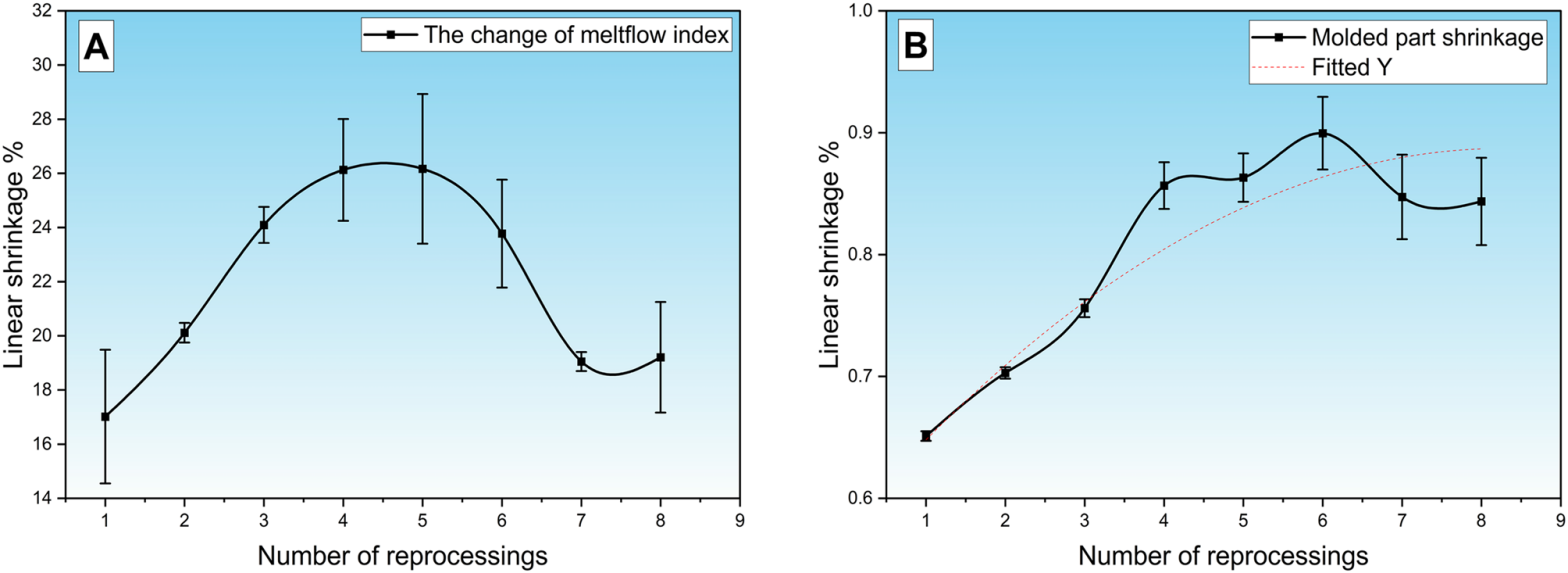
Change in MFI changes by generation (**A**) and linear shrinkage for molded green part (**B**).

To investigate the observed change in the melt flow index (MFI) over successive generations, thermogravimetric analysis (TGA) was performed to examine the decomposition behavior of the binder system. We hypothesize that the observed changes in MFI are primarily caused by a reduction in wax content, a key component in enhancing flowability. This hypothesis is supported by the TGA results (see Table 2), where the first two decomposition steps correspond to the volatilization of wax components. The decrease in wax content could lead to reduced flowability after the 4th generation, as seen in Fig. 5A. Therefore, the thermal degradation pattern observed in TGA analysis confirms that the loss of wax is a plausible explanation for the decline in MFI beyond generation 4. The linear dimensional deviation of the specimens was evaluated in the experiment, where the distance between two holes in the specimen was measured with high accuracy. Specimen shrinkage was quantified by referencing the initial dimensions of the mold cavity (Fig. 5B), and the linear shrinkage value was defined for the “green” product after injection molding. The results for the green product clearly show that shrinkage increases until generation 3, after which it stagnates. Machine parameters remained constant throughout the experiments; observed variations thus reflect intrinsic changes in the flow properties of the recycled feedstock. The crystallization process influences the shrinkage. Feedstocks with a higher flow index may cool faster, resulting in a less ordered crystal structure. A disordered structure leads to higher shrinkage as the molecules are rearranged to a greater extent during cooling. This effect may indicate a loss of wax since it increases the particle size of the metallic powder**.** The melt flow index was determined for each generation of regrind, to show that successive reprocessing increases the melt flow index of the feedstock, similar to the recycling of plastic^31^.

The series of MFI measurements demonstrates high reproducibility, initially exhibiting an upward trend until the fourth cycle, followed by a noticeable decrease from the fifth cycle onward. The maximum of the curve shows a different character from the curve typical for plastics^32^, which we hypothesize is due to the fact that during the recycling of the polymer, not only a change in viscosity due to the fragmentation of the polymer chains takes place, but also to a change in another flow aiding component, such as a decrease in wax. In a 2018 study, A. Dehghan-Manshadi and colleagues provided a detailed analysis of the changes in mechanical properties, shrinkage, and density have a significant impact. In our case, the observed variance can likely be attributed to the positioning of the sample pieces within the furnace^33^.

### Thermal analysis

**Fig. 6:**
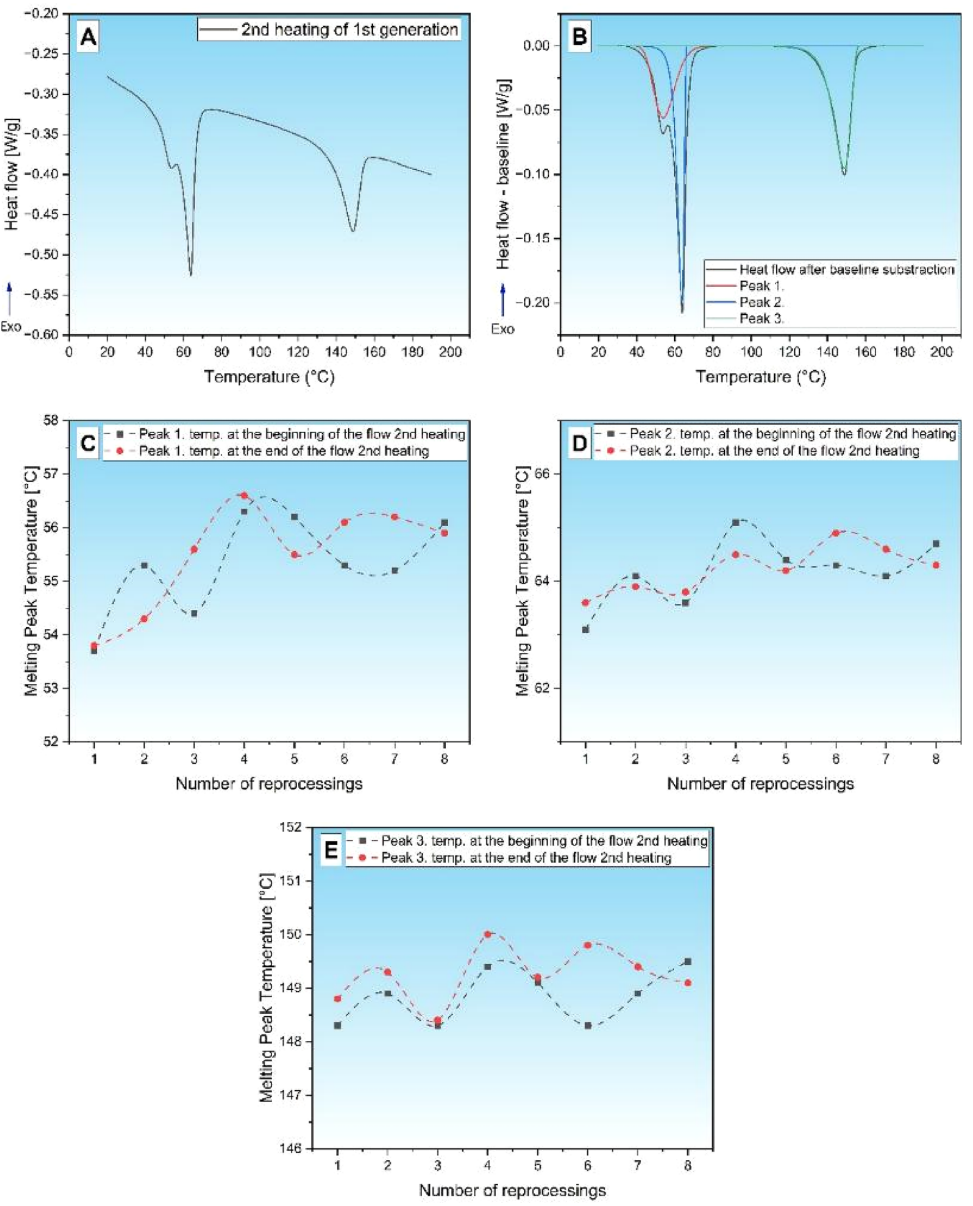
1st generation, beginning of flow path, DSC curve of 2nd heating (**A**), and peak resolution after baseline subtraction (**B**) Change in peak melting temperatures at the beginning and end of the flow path (**C**): peak 1; (**D**): peak 2; (**E**): peak 3.

The polymeric constituents of the feedstock were characterized using differential scanning calorimetry (DSC) analysis. The results of Fig. 6/A/B show the typical DSC curve shape obtained during the test. The heating curve shows three endothermic peaks. Of these, the first two overlapped in the temperature range of 35–80 °C, presumably indicating the melting of the slip agents (paraffin wax, stearate-acid)^34^, and the third one between 120–165 °C shows the melting of the PP constituent.

From this, the peak temperature of each endotherm was determined for further analysis. The peak melting temperatures at the beginning and end of the flow path of the test specimens as a function of generations are shown in Fig. 6C,D,E. It can be seen that there is no significant difference in peak temperature for either melting endotherm in the samples taken from the beginning and end of the flow path. A slight increase in peak temperature is observed for all three peaks as a function of generations, which may indicate an increase in crystallite sizes. This may indirectly signify the degradation of polymer molecular chains^35^.

**Fig. 7:**
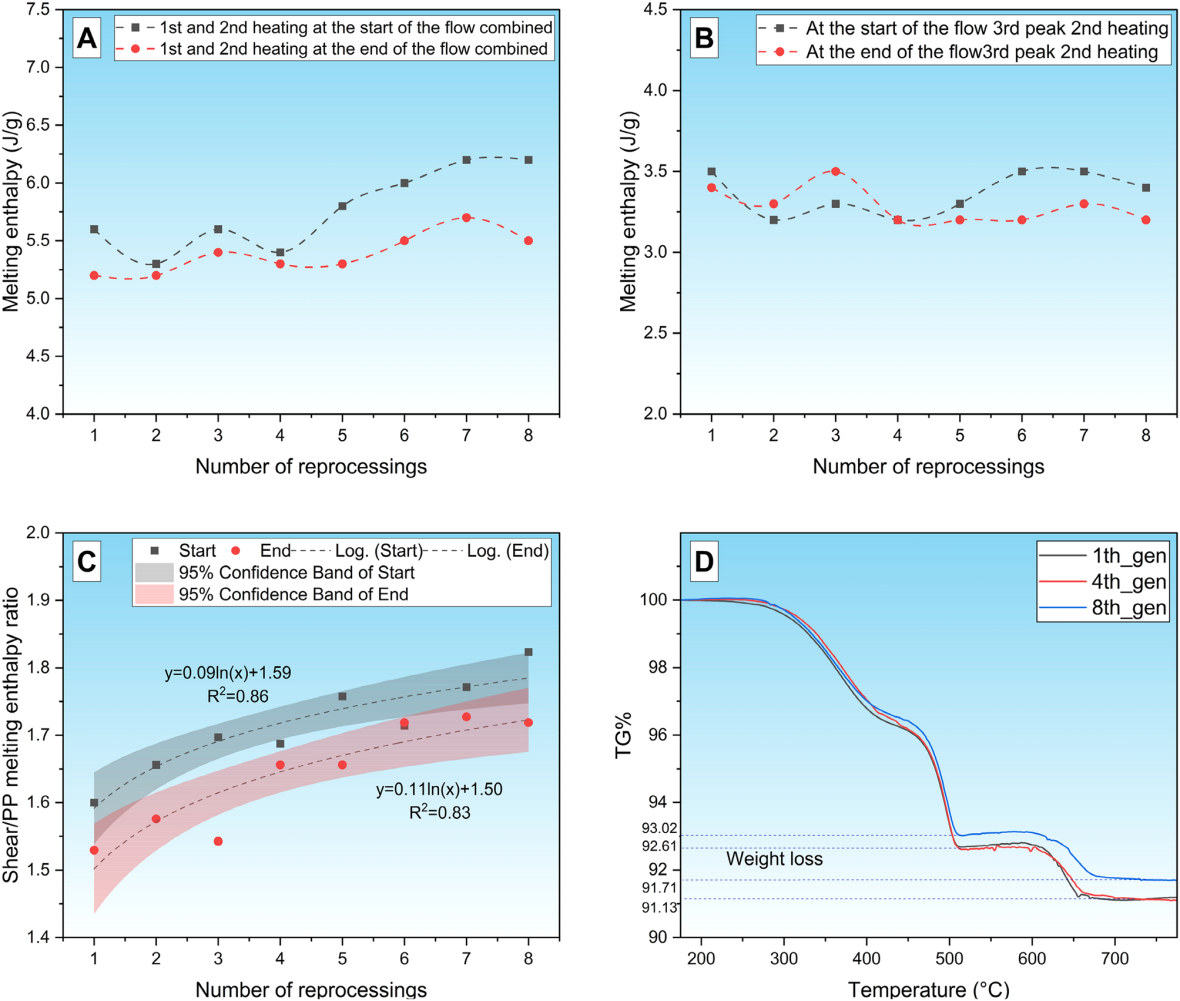
Changes in peak melting enthalpy as a function of generations (**A**) 1 + 2 peak; (**B**) peak 3) enthalpy ratio (**C**) and TGA curves of generation 1,4 and 8 samples (**D**).

Additionally, melting enthalpies were systematically evaluated to understand thermal transitions in detail. In this case, we did not separate peaks 1 and 2 belonging to the slider but examined the enthalpy of Fig. 7A,B, and its ratio to the enthalpy of the PP melting peak was explored in Fig. 7C. It can be observed that the enthalpy of peak 1 + 2, which is characteristic of sliders, shows an increasing trend with the growing number of generations, but the intensity of the increase is different near the injection point of the injection molded specimens and at the end of the flow path: it is more intense at the beginning and increases to a lesser extent at the end. The enthalpy of the melting peak for PP does not show a significant change at any of the sampling points, hence the wax/PP enthalpy ratio shows an increasing character.

There are three main reasons for the increase in enthalpy:

1. Changes its crystalline modification;
2. The proportion of the constituent in the composition increases;
3. The crystalline fraction of a given component enhances^36^.

The first case is unlikely, as peak melt temperatures have only changed slightly. The second case would imply that the proportion of PP has decreased, which is improbable because PP has higher thermal stability than waxes^37^, or a mixture has occurred, and the PP-wax ratio is not constant along the flow path. In the third case, an increasing crystalline fraction of the sample is likely, since the tendency of molecular chains to shorten during degradation to arrange is higher, which may explain the increasing enthalpy value^38^.

The increase in enthalpy of peaks 1–2 may therefore be caused by a change in the slip/PP ratio and/or a difference in the crystalline fraction of the constituents. To determine the constituent fraction, TGA was performed on generation 1, 4 and 7 samples Fig. 7D. It should be noted that the relative mass changes detected were small, as the majority of the total mass of the samples is due to metal dust loading. Four ranges of decomposition temperatures were detected on the TG curves, and the mass losses at these temperatures are summarized in Table 3. It can be observed that in the first decay range (175–250 °C), only in the 1st generation sample could a mass loss be witnessed. This suggests that a fraction of the slip agent decomposes at this temperature^39^ so this fraction is present in decreasing amounts in successive reprocessing steps and is no longer detectable in the 4^th^ generation samples. The second degradation step is likely to indicate wax degradation, while the third one is associated with PP degradation. There was only a slight difference between the generations tested. In the fourth (580–700 °C) decomposition step, a mass loss of 1.4–1.7% was observed.

**Table 3:** Decomposition steps determined from the TGA test.

Weight loss (%)	1. decomp. stage (175–250 °C)	2. decomp. stage (250–450 °C)	3. decomp. stage (450–525 °C)	4. decomp. stage (580–700 °C)
1. Generation	0.2	3.7	3.4	1.7
4. Generation	0.0	3.7	3.5	1.5
8. Generation	0.0	3.6	3.5	1.4

### Evaluation of composition ratio variations using SEM and EDS analysis

**Fig. 8:**
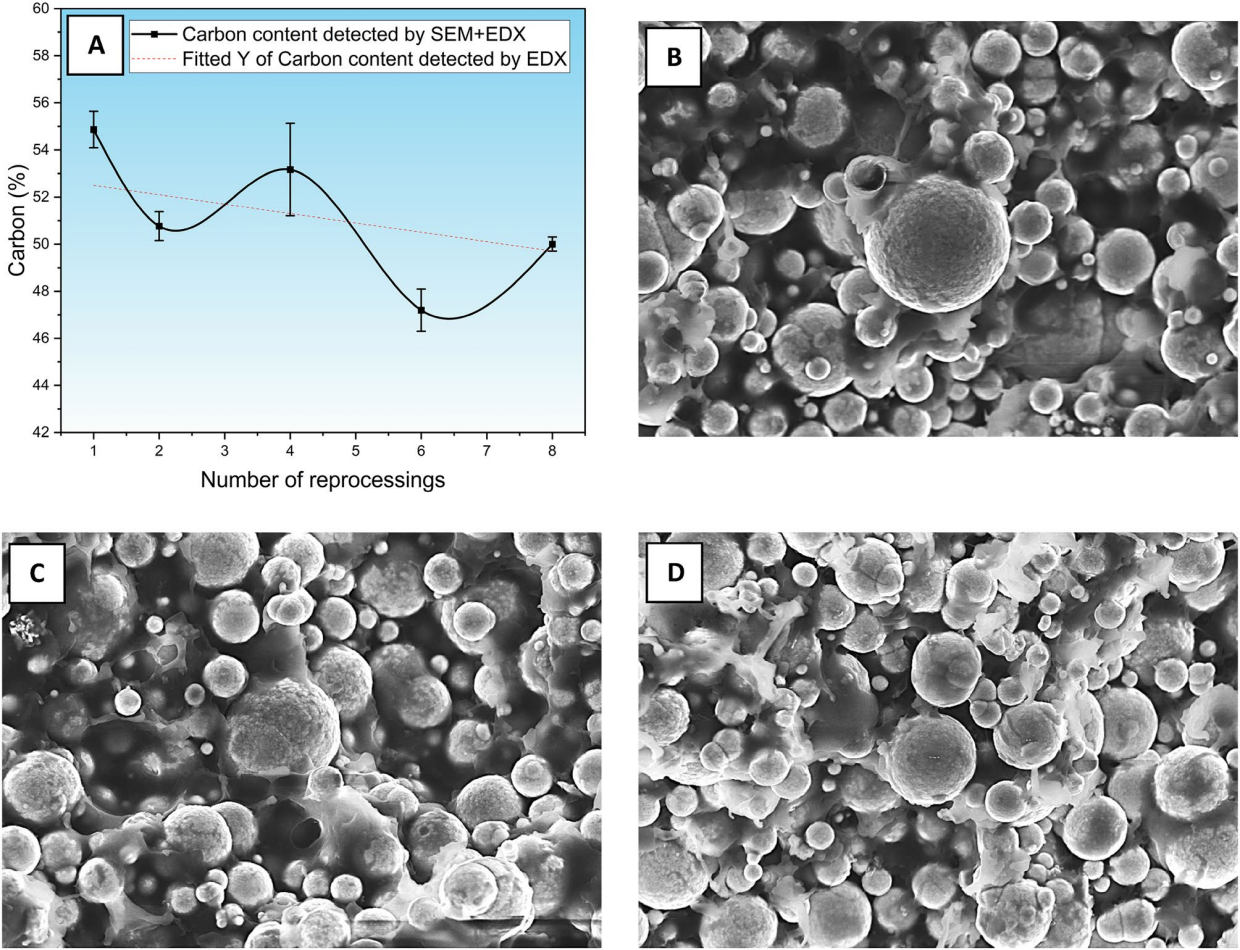
Changing carbon content by generation (**A**), SEM Image (10Kx) Gen_1 green state (**B**), SEM Image green state (10Kx) Gen_4 (**C**), SEM Image green state (10Kx) Gen_8_green state (**D**).

Sample evaluations were substantiated by detailed scanning electron microscopy (SEM) analysis. As in previous studies, the images were taken of green samples still containing binders. In the images obtained, the metal particles, their distribution and size distribution are visible, and the embedding of the particles in the aggregate is very well observed. Figure 8B shows the first generation, Fig. 8C shows the fourth generation and Fig. 8D the eighth generation at a magnification of 10000x. It is important to note that the scanned area is the burr surface from the test section of the tensile specimens. Based on the original assumption that the binder system is changed by recycling, it should be possible to detect the composition as expected by the electron microscope and detected by the EDS detector. So, if the assumption is true, the reduction in the binder content of the wax leaving the sum of the waxes during the injection molding process should cause a decrement in the carbon content of the scanned surface. The working distance between the workpiece and the electron gun exit was increased to 8.5 mm to detect a larger backscatter.

To obtain more accurate results, 3 different locations were scanned and excavated from each sample tested, but not all generations were tested. Furthermore, it is vital to note that the scanning gives an average of 10–15 thousand particles and bedding material. Six elements known to be constituents of the components carbon (C), oxygen (O), silicon (Si), iron (Fe), nickel (Ni), and molybdenum (Mo) were measured. Since elements that were not part of a component were rarely present in the mixture, the evaluation was focused exclusively on the six elements mentioned above.

The proportion of detected elements can be evaluated according to Table 4:

**Table 4:** Quantity of elements detected by EDS detector.

Element	C	O	Si	Fe	Ni	Mo
Weight %	49.7	1.9	0.3	47.3	0.5	0.3

The results of the different generations are plotted on a graph, which illustrates the variation of carbon content via generation Fig. 8A. A distinct decrease in carbon content is observed from 55 to 50% as a result of reprocessing. Since the scanning is for a specific area, the weight percentages of the materials used are 55% metallic powder and 45% binder. Most of the binder is composed of carbon compounds, and the metallic dust itself contains a small amount of carbon (0.5%). The results show that the carbon content is not entirely in line with expectations, which is assumed to be caused by the frequent presence of binder on the surface of the grains, as indicated by Fig. 7B,C and D. The 5% reduction in the measured carbon content (from 55 to 50%) does not directly translate to a 5% wax loss. Given that the majority of the carbon originates from the wax fraction of the binder, the actual wax loss is likely to be significantly higher. This relationship can be approximated using the following expression:

$$Wax loss \left[\%\right]=\frac{{C}_{0}-{C}_{1}}{{C}_{0}}\cdot \frac{1}{{f}_{c,wax}}\cdot 100$$

where C_0_​ and C_1_ are the initial and measured carbon contents from EDS analysis, and f_C,wax_ is the estimated fraction of total carbon attributed to the wax component. Assuming f_C,wax_ = 0.45, the estimated wax loss is approximately 20.2%.

In his 2020 study, Kayping Yu demonstrated that an additional dose of graphite improved the mechanical properties of the parts to a certain extent, interacting with oxide formation. Therefore, it makes sense that a reduction in carbon content leads to increased oxide formation, which deteriorates the mechanical properties.^40^

## Conclusion

A comprehensive analysis of the raw material was conducted through multiple reprocessing cycles, revealing the evolving structural and thermal behavior of polymer binders in MIM technology. After the initial injection molding, the material underwent seven consecutive recycling steps, during which its mechanical, thermal, and rheological properties were systematically evaluated. Advanced characterization techniques, including SEM analysis, MFI measurements, dimensional shrinkage evaluation, DSC, and TGA, provided critical insights into the material’s degradation pathways and processability limits.

The findings confirm that polymer binder degradation and wax precipitation play a crucial role in determining the recyclability of MIM feedstocks. The melt flow index measurements indicate that the most significant changes occur by the fourth recycling cycle, beyond which material viscosity and processability begin to decline. The crystallization behavior of the polymer matrix, as inferred from DSC and shrinkage analysis, suggests that progressive molecular fragmentation leads to increased crystallite size and altered viscosity. This effect is counterbalanced by the reduction in wax content, which influences the overall thermal stability and rheological performance of the feedstock.

TGA results identified distinct decomposition stages, with early-stage material loss attributed to slip agents and wax degradation, while polypropylene decomposition exhibited a relatively stable trend over multiple cycles. Scanning electron microscopy and EDS analysis further validated these observations, revealing a measurable decrease in carbon content, indicative of progressive wax loss. These microstructural changes directly impact the material’s flow characteristics, emphasizing the importance of binder optimization strategies for extended recyclability.

From an engineering and sustainability perspective, these findings highlight the potential for integrating multi-cycle polymer binder reuse into MIM manufacturing workflows. The ability to maintain thermal and flow properties over four recycling cycles demonstrates the viability of resource-efficient material design in metal injection molding. This study underscores the need for further investigation into binder system modifications and additive strategies that could enhance long-term recyclability without compromising material integrity. While the present work did not include any changes to the binder formulation or processing parameters, our findings suggest that such optimization approaches—particularly in terms of component selection and thermal profile adjustments—may be beneficial. By addressing these challenges, MIM technology can contribute to circular economy initiatives, reducing raw material dependency while ensuring high-performance component manufacturing.

## Data Availability

Data availability All data generated or analysed during this study are included in this published article.
